# T-Cell Autophagy Deficiency Increases Mortality and Suppresses Immune Responses after Sepsis

**DOI:** 10.1371/journal.pone.0102066

**Published:** 2014-07-16

**Authors:** Chih-Wen Lin, Steven Lo, Chin Hsu, Chi-Hsun Hsieh, Ya-Fang Chang, Bao-Sheng Hou, Ying-Hsien Kao, Chih-Che Lin, Ming-Lung Yu, Shyng-Shiou Yuan, Ya-Ching Hsieh

**Affiliations:** 1 Division of Gastroenterology and Hepatology, Department of Medicine, E-Da Hospital, I-Shou University, Kaohsiung, Taiwan; 2 Department of Plastic and Reconstructive Surgery, E-Da Hospital, I-Shou University, Kaohsiung, Taiwan; 3 Department of Medical Research, E-Da Hospital, I-Shou University, Kaohsiung, Taiwan; 4 Health Examination Center, E-Da Hospital, I-Shou University, Kaohsiung, Taiwan; 5 Canniesburn Plastic Surgery Unit, Royal Infirmary, Glasgow, United Kingdom; 6 Graduate Institute of Medicine, College of Medicine, Kaohsiung Medical University, Kaohsiung, Taiwan; 7 Department of Physiology, Faculty of Medicine, College of Medicine, Kaohsiung Medical University, Kaohsiung, Taiwan; 8 Hepatobiliary Division, Department of Internal Medicine, Kaohsiung Medical University Hospital, Kaohsiung, Taiwan; 9 Translational Research Center and Cancer Center, Kaohsiung Medical University Hospital, Kaohsiung, Taiwan; 10 Department of Obstetrics & Gynecology, Kaohsiung Medical University Hospital, Kaohsiung, Taiwan; 11 Department of Surgery, China Medical University Hospital, China Medical University, Taichung, Taiwan; 12 Department of Surgery, Benq Medical Center at Suzhou, Suzhou, China; 13 Division of General Surgery, Department of Surgery, Kaohsiung Chang Gung Memorial Hospital, Kaohsiung, Taiwan; 14 College of Medicine, Chang Gung University, Gueishan, Taiwan; University of São Paulo, Brazil

## Abstract

**Background:**

Although the role of autophagy in sepsis has been characterized in several organs, its role in the adaptive immune system remains to be ascertained. This study aimed to investigate the role of autophagy in sepsis-induced T cell apoptosis and immunosuppression, using knockout mice with T cell specific deletion of autophagy essential gene *Atg7*.

**Methods and Results:**

Sepsis was induced in a cecal ligation and puncture (CLP) model, with T-cell-specific *Atg7*-knockout mice compared to control mice. Autophagic vacuoles examined by electron microscopy were decreased in the spleen after CLP. Autophagy proteins LC3-II and ATG7, and autophagosomes and autolysosomes stained by Cyto-ID Green and acridine orange were decreased in CD4^+^ and CD8^+^ splenocytes at 18 h and 24 h after CLP. This decrease in autophagy was associated with increased apoptosis of CD4^+^ and CD8^+^ after CLP. Moreover, mice lacking *Atg7* in T lymphocytes showed an increase in sepsis-induced mortality, T cell apoptosis and loss of CD4^+^ and CD8^+^ T cells, in comparison to control mice. This was accompanied by suppressed cytokine production of Th1/Th2/Th17 by CD4^+^ T cells, reduced phagocytosis in macrophages and decreased bacterial clearance in the spleen after sepsis.

**Conclusion:**

These results indicated that sepsis led to down-regulation of autophagy in T lymphocytes, which may result in enhanced apoptosis induction and decreased survival in sepsis. Autophagy may therefore play a protective role against sepsis-induced T lymphocyte apoptosis and immunosuppression.

## Introduction

Despite advances in the care of critically ill patients, sepsis leading to multiple organ failure still remains the major cause of death in severely injured patients who survive initial trauma, hemorrhage, or burn injury [1], [2], [3], [4]. Sepsis is characterized by an initial hyper-inflammatory response, followed by a period of immunosuppression termed “immunoparalysis” [5]. Increased lymphocyte apoptosis has been correlated with decreased survival in experimental animal studies, and confirmed in observational human studies [6], [7], [8]. As lymphocytes produce proinflammatory cytokines and activate macrophages, loss of lymphocytes can impair the ability of the immune system to combat pathogens [8], [9]. Investigating the role and mechanisms of lymphocyte death may develop new effective strategies in the treatment of sepsis.

Autophagy plays a protective role in liver, heart, lung and kidney in sepsis, which protects against apoptotic cell death [10], [11], [12], [13], [14], [15]. Autophagy also has critical physiological functions in the immune system. In the absence of the autophagy-related genes - *Atg7* or *Atg5*, both CD4^+^ and CD8^+^ T lymphocytes rapidly undergo apoptosis in the periphery, with reduced numbers in secondary lymphoid organs, and a failure to proliferate in response to T cell antigen receptor stimulation [16], [17]. Autophagy also affects the presentation of cytosolic antigens in the context of MHC II molecules [18], in T-cell development, differentiation, polarization, and homeostasis [19], [20]. Moreover, autophagy is one of the main degradative pathways of the immune system responsible for the detection of intracellular bacteria, such as *Mycobacterium tuberculosis* and *Streptococcus pyogenes* [21], [22], [23]. In addition, autophagy cooperates with Toll-like receptors, and acts as both a regulator in plasmacytoid dendritic cells response to viral infection [24].

The autophagy process is initiated by the formation of an isolation membrane in cytoplasm. Through an elongation step, the isolation membrane forms a double-membrane structure called the autophagosome to encapsulate cytoplasmic contents, damaged organelles, or invading intracellular pathogens. The autophagosome then fuses with the lysosome for the degradation of the encapsulated materials [25]. Autophagy ATG7 is an essential protein required for the elongation phase of autophagosome formation [26], [27], [28]. Therefore in this study we employed a mouse with *Atg7* knockout specific to T lymphocytes only, in order to determine the role of autophagy in regulating T lymphocyte apoptosis and immune responses in sepsis.

## Materials and Methods

### Ethics statement

Animal experiments were performed in strict accordance with the international guidelines for the care and use of laboratory animals and with ethics approval from the Institutional Animal Care and Use Committee (IACUC) of E-Da Hospital/I-Shou University, Taiwan (Permit number: IACUC-100010).

### Animals

Experiments were performed on male mice (6-8 weeks old). C57BL/6 mice (BioLASCO Taiwan Co., Ltd., Taipei, Taiwan) were used for time-point studies. Transgenic Atg7^floxp/floxp^ mice (loxP-*Atg7* conditional targeting allele, referred to as Atg7^f/f^) were obtained from Dr. Masaaki Komatsu, Laboratory of Frontier Science, Tokyo Metropolitan Institute of Medical Science, Bunkyo-ku, Tokyo, Japan. Transgenic CD4-Cre mice that express Cre-recombinase under the control of the lck proximal promoter or CD4 enhancer/promoter/silencer were obtained from European Mouse Mutant Archive [29] (Monterotondo, Italy). To generate mice with Atg7-deficient T lymphocytes, Atg7^f/f^ mice (on a pure C57BL/6 background) were crossed to transgenic CD4-Cre (backcrossed for 6 generations onto the C57BL/6 background). This generated doubly transgenic mice (Atg7^f/f^CD4-Cre) in which the *Atg7* gene was deleted by Cre recombinase expression in T cells. Thus, Atg7^f/f^ mice were used as the control mice and doubly transgenic Atg7^f/f^CD4-Cre mice were used as the T cell-specific *Atg7* deletion mice (6–7 weeks old). All the mice were kept in the animal center of I-Shou University at a controlled temperature of 22±1°C, relative humidity 55±5%, and with 12 h light/12 h dark cycles for 1 week before the experiment.

### Sepsis model

Sepsis was induced by cecal ligation and puncture (CLP) as described previously [30], [31]. Briefly, under isoflurane anesthesia (2%), the cecum was exposed by a 1-cm midline laparotomy and was ligated below ileocecal junction. Two cecal punctures were made with a 22-gauge needle and a small amount (droplet) of feces was pressed out to ensure patency of the punctures. The bowel loops were returned to their anatomical position and the abdominal wall was closed in layers using 6-0 surgical sutures (Ethicon Inc., Somerville, NJ). Postoperatively, 1 ml of 0.9% saline was administered subcutaneously. Before and after the surgery, animals had unrestricted access to food and water. Sham-operated mice were operated identically, except that the cecum was not ligated or punctured. Six, twelve, eighteen and twenty-four hours after surgery, the animals were killed, and splenocytes were isolated for further evaluation. The animals were euthanized with isoflurane anesthesia at the end of experiments. All surgery was performed under isoflurane anesthesia, and all efforts were made to minimize suffering.

In the survival study, mice following CLP were allowed access to food and water ad libitum and monitored every 6 h for 7 days. Moribund animals were identified by labored breathing and/or non-responsiveness to cage tapping. Moribund mice were humanely sacrificed by using CO_2_ followed by cervical dislocation. At the end of the study (day 7), all the surviving mice were euthanatized with CO_2_ followed by cervical dislocation.

### Transmission electron microscopy

Spleen tissues were excised and fixed with fixative buffer containing 2% paraformaldehyde and 2.5% glutaraldehyde in PBS and were stored at 4°C until embedding. Tissue samples were then post-fixed in 1% phosphate-buffered osmium tetroxide and embedded in Spurr's resin. Sections were cut 0.12-µm thin and stained with 0.2% lead citrate and 1% uranyl acetate. Images were examined with a JEOL TEM-2000 EX II at 80 kV (Tokyo, Japan). Specimens were examined as previously described [10]. Briefly, three sections from each block were chosen at random for ultrastructural measurement. Thirty non-repeating micrographs (at 7,500×) per animal were captured in randomly selected fields. The area of one micrograph was regarded as the unit area. The numbers of autophagic vacuoles per unit area were counted.

### Splenocyte preparation

Spleens were placed in ice cold, 4°C PBS and gently ground between frosted slides to produce a single-cell suspension. The suspension was centrifuged at 400×*g* for 10 min and the pellet was resuspended in PBS. Red blood cells were lysed with erythrocyte lysis buffer (BD Pharmingen, San Diego, CA) and the remaining cells were washed with PBS by centrifugation at 400×*g* for 10 min. Cell viability was consistently >95%, as determined using trypan blue exclusion procedure. CD4^+^ or CD8^+^ cells were purified by positive selection using CD4 or CD8 microbeads (>95% purity) obtained from Miltenyi Biotec (Bergisch Gladbach, Germany). For cytokine production, purified CD4^+^ lymphocytes were cultured on plates coated with antibodies to 1 µg/ml of CD3 (145-2C11; BD Pharmingen) and 1 µg/ml of CD28 (37.51; BD Pharmingen) in RPMI 1640 medium (Invitrogen, Carlsbad, CA) with 10% heat-inactivated FBS (Invitrogen) at 37°C, 95% humidity, and 5% CO_2_ for 24 h. After incubation, the cell-free suspension was collected and stored at −80°C until further analysis.

### Western blot analysis

CD4+ and CD8+ cells were purified by positive selection using CD4 and CD8 microbeads obtained from Miltenyi Biotec. Sample preparation and Western blotting were carried out as previously described [15], [32]. Membranes were immunoblotted with microtubule-associated protein light chain (LC3), ATG7 (Novus Biologicals, Littleton, CO), and Actin (a loading control)(BD Pharmingen) antibodies.

### Cell-surface marker staining

Cells (1×10^6^ cells/ml) were washed twice with Stain Buffer (BD Pharmingen) and resuspended in 50 µl Stain Buffer (BD Pharmingen) containing surface markers PE Cy7-anti-CD4 (BD Pharmingen), PC5.5-anti-CD8 (Beckman Coulter, Brea, CA), or appropriate isotype control was added. After 30 min on ice, cells were washed twice with Stain Buffer. These cells were then stained with Acridine orange, Cyto-ID Green, TUNEL, or Annexin-V staining (as below) and analyzed by flow cytometry (Beckman-Coulter FC-500 Analyzer) using Beckman-Coulter Kaluza software. CD4^+^ and CD8^+^ cells were determined after gating on a population of lymphocytes in which 3×10^4^ to 5×10^4^ cells per sample were counted and processed for Acridine orange, Cyto-ID Green, TUNEL, or Annexin-V assays.

### Quantification of absolute cell numbers

The absolute cell count for each population subset was calculated by the following formula: cell counts of cell subpopulations  =  total cell counts multiplied by the subset population percentage.

### Acridine orange staining

Acridine orange (AO) is used in autophagy assays and stains autolysosomes. Cells were incubated with medium containing 0.5 µg/ml acridine orange (Sigma-Aldrich, St. Louis, MO) for 30 min at 37°C and then washed once with PBS to remove acridine orange. It crosses into lysosomes (and other acidic compartments) and becomes protonated. The protonated dye stacks and stacked acridine orange emits in the red range determined by flow cytometry (Beckman-Coulter) using Beckman-Coulter Kaluza software.

### Cyto-ID Green staining

Cyto-ID Green Detection Reagent Kit (Enzo Life Sciences, Farmingdale, NY) detects autophagosomes and autolysosomes as recommended by manufacturer [33]. In brief, cells were stained with Cyto-ID Green for 30 minutes at 37°C and the stained cells were immediately analyzed via flow cytometry (Beckman Coulter) using Beckman-Coulter Kaluza software.

### Annexin-V staining

Cell apoptosis was analyzed by Annexin-V-FITC (BD Pharmingen) according to the manufacturer's instructions. In brief, cells were resuspended in binding buffer containing Annexin-V for 15 min at room temperature. The cells were then diluted by adding 300 µl of binding buffer and analyed by flow cytometer using Kaluza software (Beckman Coulter).

### TUNEL staining

Cell apoptosis was also determined by a terminal deoxynucleotidyl transferase (TdT)-mediated dUTP nick end labelling (TUNEL) assay kit (In Situ Cell Death Detection Kit-Fluorescein; Roche Molecular Biochemicals, Temecula, CA) according to the manufacturer's instructions. In brief, cells were fixed with 2% paraformaldehyde in PBS (pH 7.4) at 4°C for 1 h and wash twice by PBS then resuspended in 0.1% sodium citrate containing 0.1% Triton X-100 for 1 h. The cells were then treated with TUNEL reaction mixture containing terminal deoxynucleotidyl transferase (TdT) and fluorescein-dUTP, and the cells were incubated at 37°C in a humidified atmosphere for 1 h. After incubation, cells were washed twice by 1 ml Rinse buffer and add 0.5 ml PBS. The cells were analyzed with a flow cytometer using Kaluza software (Beckman Coulter).

### Cytokine analysis

The concentrations of cytokines were measured by commercially available cytometric bead array (CBA) Mouse Th1/Th2/Th17 Cytokine kit (BD Biosciences, San Jose, CA), according to the manufacturer's instructions. Briefly, 50 µl of mixed capture beads were incubated with 50 µl of plasma samples for 1 h at 25°C and then 50 µl of mixed PE detection reagent was added. The immunocomplexes were then washed and analyzed using a BD Biosciences FACSCalibur flow cytometer (BD Biosciences). Data analysis was carried out using the accompanying CellQuest Pro and FCAP Array software (BD Biosciences).

### Phagocytosis

pH-sensitive pHrodo-conjugated *E. coli* BioParticles (Invitrogen) were used to detect the acidity of the phagosome upon internalization. Splenocytes were incubated in RPMI 1640 complete medium (Invitrogen) containing 1 mg/ml of pHrodo-conjugated *E. coli* BioParticles at 37°C, 95% humidity and 5% CO_2_ for 1 h. Cells were then harvested and stained with macrophage marker APC-anti-F4/80 (BD Pharmingen) and subjected to flow cytometry analysis. The cell populations gated as F4/80-positive were analyzed for phagocytosis.

### Bacteria loads in blood and spleen

Whole blood was collected and diluted with sterile PBS. Spleen tissues were removed and mechanically homogenized with 1 ml PBS under sterile conditions. Samples were aseptically spread on tryptic soy agar plates (Creative Media Products, Taipei, Taiwan). The plates were incubated at 37°C aerobically for 24 h. At the end of the incubation period, colony forming units (CFU) were counted.

### Statistical analysis

Data are represented as mean ± SEM. Statistical analysis of the data was performed by using one-way analysis of variance followed by Student-Newman-Keul's test. The comparison between two groups was analyzed by two-tailed Student t-test. Survival analyses were performed by Kaplan-Meier analysis. *P*<0.05 was considered to be statistically significant.

## Results

### Electron microscopic characterization of autophagic vacuoles after CLP

**Figure 1:**
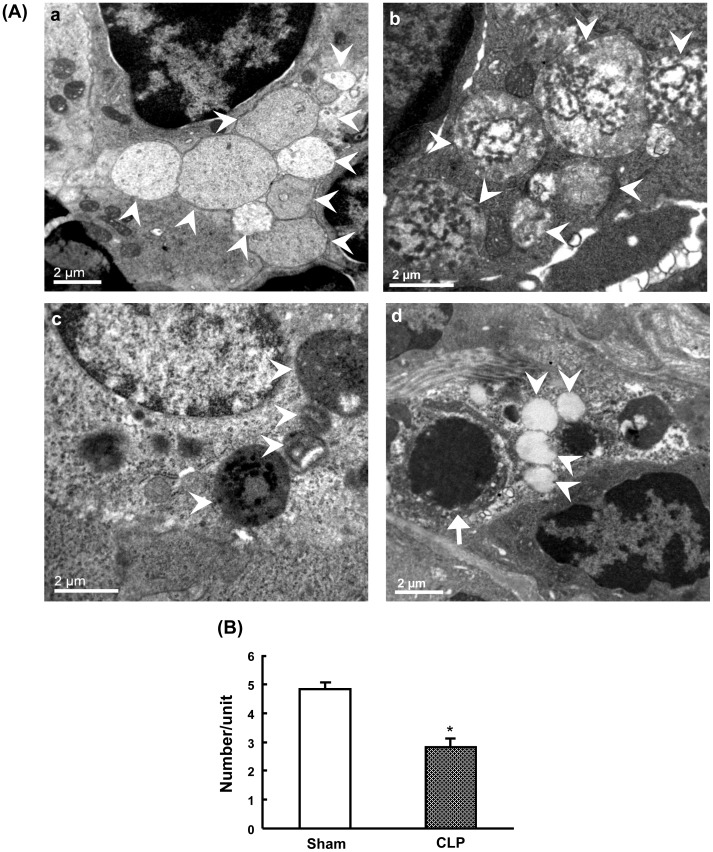
Ultrastructural features of autophagic vacuoles after CLP. Autophagy was mophologically characterized by transmission electron microscopy (A). Splenic tissues were harvested at 24h after CLP. In sham mice (a and b), splenocytes were normal in appearance with proper mitochondria distribution. Sham mice revealed autophagic vacuoles (arrowheads) in the cytosol with double- or single-membrane structures containing digested cytoplasmic components. CLP mice (c and d) displayed less autophagic vacuolization. A representative cell showed apoptosis in CLP mice with cell shrinkage, nuclear condensation and cellular disorganization (d, arrow). Quantification of autophagic vacuoles in sham and CLP mice (B). The number of autophagic vacuoles was counted under the microscope at 7,500× from 30 non-repeating micrographs for each mouse. Data are shown as mean ± SEM of 3 animals in each group and compared by two-tailed Student t-test. *P<0.05 vs. sham-operated mice. CLP: cecal ligation and puncture.

Transmission electron microscopy was performed for qualitative and quantitative characterization of autophagy in the spleen following CLP induced sepsis. Firstly, sham-operated mice revealed autophagic vacuoles in the cytosol with double- or single-membrane structures containing digested cytoplasmic components (**Figure 1A** **a and 1Ab**). At 24 h after CLP, splenocytes revealed a decreased number of vacuolization compared to sham-operated animals. Most of these vacuoles were surrounded by single-membrane structures (**Figure 1A** **c and 1Ad**). The quantitative results showed a significant decrease in autophagic vacuoles at 24 h after CLP compared to sham-operated mice (**Figure 1B**).

### Decreased autophagy is associated with increased apoptosis after CLP

**Figure 2:**
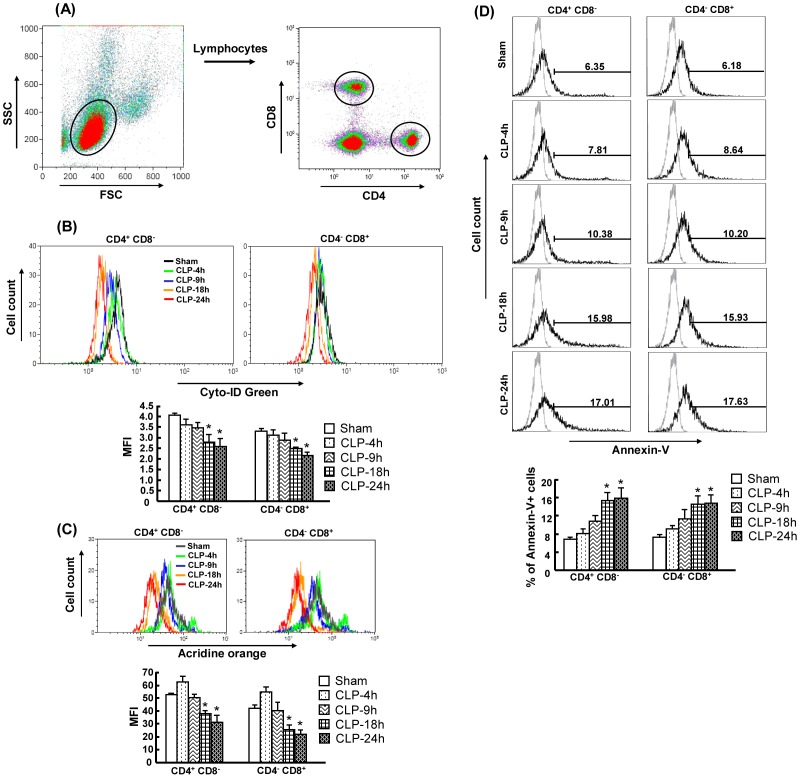
Decreased autophagy and increased apoptosis in CD4^+^CD8^−^ and CD4^−^CD8^+^ T cells after CLP. Spenocytes were obtained at 4h, 9h, 18h and 24h after CLP. Gating strategy for CD4^+^CD8^−^ and CD4^−^CD8^+^ T cells by flow cytometry analysis (A) Cells were initially gated on a forward- and side-sactter lymphocytes gate to exclude dead cells, monocytes and granulocytes. The gated lymphocytes were then selected for either CD4^+^CD8^−^ and CD4^−^CD8^+^ T cells. Cyto-ID Green (B), acridine orange (C) and Annexin-V (D) staining were further analyzed by flow cytometry. Representative histograms were gated on CD4^+^CD8^−^ and CD4^−^CD8^+^ T cells. Values were shown as mean fluorescence intensities (MFI) for Cyto-ID Green/acridine orange and percentage for Annexin-V staining. In the histogram of Annexin-V staining, light gray histogram represents a staining control without adding Annexin-V. Results obtained from 5–6 animals in each group are shown as mean ± SEM in the bar graph. Data are compared by one-way analysis of variance and Student-Newman Keul's test. CLP: cecal ligation and puncture. *P<0.05 vs. sham-operated mice.

To further study whether autophagy is involved in apoptosis during sepsis, we evaluated the time-course of autophagy and apoptosis in CD4^+^CD8^−^ and CD4^−^CD8^+^ splenic T cells after CLP. Please note that the CD4^+^CD8^−^ and CD4^−^CD8^+^ T cells were not selected by anti-CD3 surface maker. Thus, CD4^+^CD8^−^ and CD4^−^CD8^+^ T cells that mainly represent T cells, were used throughout the whole study (hereafter referred to simply as CD4^+^CD8^−^ and CD4^−^CD8^+^ T cells). Cyto-ID Green autophagy detection Kit and Acridine orange were used for detecting autophagosomes and autolysosomes. Annexin-V was used for staining apoptotic cells. Splenocytes were stained with the above dyes and then analyzed by flow cytometry. The time-course of the CLP induced septic model has been characterized to have at least two phases, a hyperdynamic phase (i.e., 4 h and 9 h after CLP) followed by a hypodynamic phase (i.e., 18 h and 24 h after CLP). Thus, CD4^+^CD8^−^ and CD4^−^CD8^+^ T cells were examined at 4 h, 9 h, 18 h and 24 h after CLP. **Figure 2A** depicts the gating strategy and an example of the gating on a positive population for CD4^+^CD8^−^ and CD4^−^CD8^+^ T cells. The results showed that the intensity of Cyto-ID Green and acridine orange was significantly decreased in CD4^+^CD8^−^ and CD4^−^CD8^+^ T cells at 18 h and 24 h after CLP compared to sham-operated mice (**Figure 2B and 2C**). The intensity of acridine orange staining was slightly but not significantly increased at 6 h after CLP in CD4^+^CD8^−^ and CD4^−^CD8^+^ T cells compared to sham-operated mice (**Figure 2C**). Moreover, the percentage of Annexin-V+ cells showed a significant increase in CD4^+^CD8^−^ and CD4^−^CD8^+^ T cells at 18 h and 24 h after CLP compared to sham-operated mice (**Figure 2D**). These results suggest that inhibition of autophagy is associated with increased apoptosis after CLP.

### Inhibition of LC3-II and ATG7 levels after CLP

**Figure 3:**
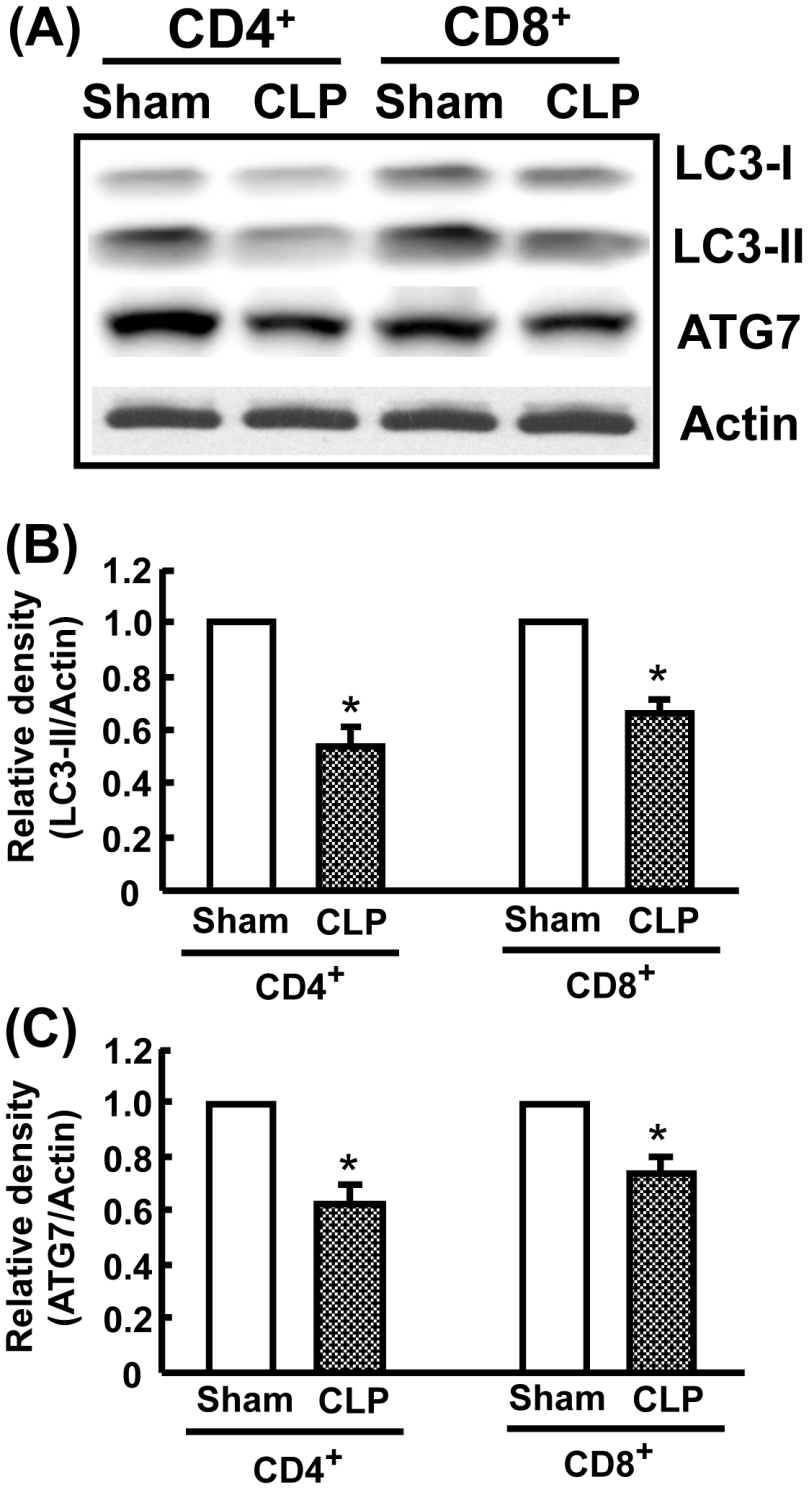
Suppression of LC3-II and ATG7 protein levels in CD4^+^CD8^−^ and CD4^−^CD8^+^ T cells after CLP. Splenic CD4^+^ and CD8^+^ T cell extracts were used for LC3-II and ATG7 protein expressions by Western blot analysis. CD4^+^ and CD8^+^ T cells obtained at 24h after CLP were isolated using CD4 and CD8 microbeads, respectively. A, Representative immunoblots of LC3-II and ATG7. B, Densitometric values of LC3-II. C, Densitometric values of ATG7. Actin was used as a loading control. Data are shown as mean ± SEM of 3 animals in each group and compared by two-tailed Student t-test. *P<0.05 vs. sham-operated mice.

LC3-II and ATG7 are specifically involved in autophagosome formation [15], [25]. We further identified the expression levels of LC3-II and ATG7 by lysate proteins extracted from CD4^+^ and CD8^+^ T cells at 24 h after CLP, as autophagy detected by Cyto-ID Green/acridine orange staining was inhibited at the late stage of sepsis. CD4^+^ and CD8^+^ cells were isolated with CD4 and CD8 MicroBeads, respectively. As shown in **Figure 3A–3C**, both LC3-II and ATG7 levels in CD4^+^CD8^−^ and CD4^−^CD8^+^ T cells were significantly deceased after CLP compared to sham-operated mice. These results correlate with the findings of Cyto-ID Green, acridine orange staining, and TEM analysis (**Figure 1** **and** **2**).

### A further decrease in autophagy in T cell-specific Atg7-knockout mice after CLP

**Figure 4:**
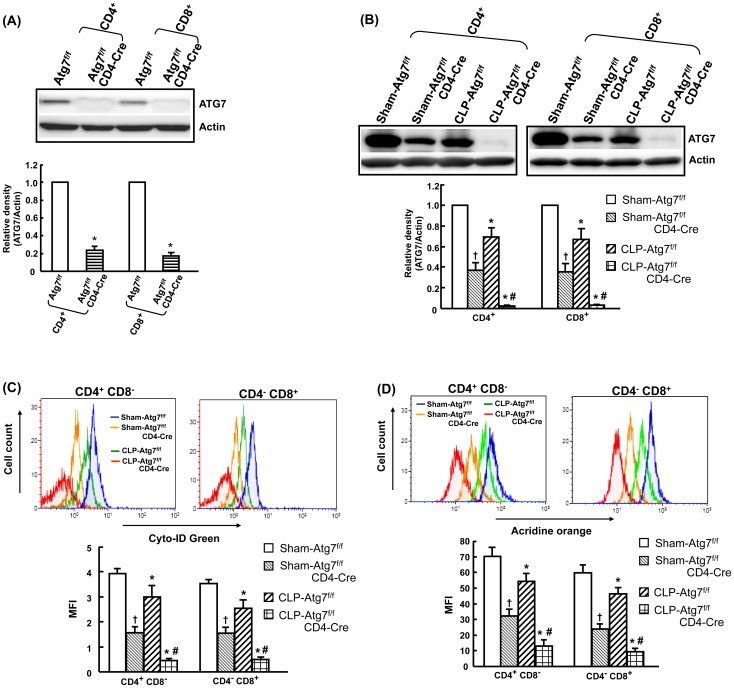
Efficacy of autophagy inhibition in Atg7^f/f^CD4-Cre mice after CLP. Atg7^floxp/floxp^ mice (Atg7^f/f^) were crossed with CD4-Cre transgenic mice to generate doubly transgenic mice (Atg7^f/f^CD4-Cre) in which *Atg7* gene was specifically deleted in T cells. ATG7 protein levels in Atg7^f/f^ and Atg7^f/f^CD4-Cre mice were detected by Western blot analysis. Splenic CD4^+^ and CD8^+^ T cells were isolated using CD4 and CD8 MicroBeads, respectively. The efficacy of protein deletion of ATG7 from CD4^+^ and CD8^+^ cells was determined in normal Atg7^f/f^ and Atg7^f/f^/CD4-Cre mice (A). ATG7 protein levels were further detected in sham and CLP-induced Atg7^f/f^CD4-Cre and Atg7^f/f^ mice (cells obtained at 18h after CLP) (B). Actin was used as a loading control. Autophagy was further evaluated by Cyto-ID Green (C) and acridine orange (D) staining and followed by flow cytometry analysis. Cells were initially gated on a forward- and side-sactter lymphocytes gate to exclude dead cells, monocytes and granulocytes. T cells were divided into CD4^+^CD8^−^ and CD4^−^CD8^+^. Representative histograms were gated on CD4^+^CD8^−^ and CD4^−^CD8^+^ T cells, and shown as mean fluorescence intensities (MFI) of Cyto-ID Green and acridine orange staining. Data are shown as mean ± SEM of 3 animals in each group and compared by two-tailed Student t-test. CLP: cecal ligation and puncture. †*P*<0.05 vs. sham-operated Atg7^f/f^ mice. *P<0.05 vs. respective sham mice. #*P*<0.05 vs. CLP-induced Atg7^f/f^ mice.

To determine whether autophagy in T cells contributes to survival in sepsis, we crossed Atg7^f/f^ mice [34] with mice expressing the T cell-specific Cre transgene (CD4-Cre mice) [29]. This generates doubly transgenic mice (Atg7^f/f^CD4-Cre) - T cell-specific knockout of *Atg7* gene. Atg7^f/f^ mice were used as the control group. Firstly, the efficiency of Cre-mediated deletion of ATG7 from splenic CD4^+^ and CD8^+^ cells was determined in normal Atg7^f/f^ and Atg7^f/f^/CD4-Cre mice. CD4^+^ and CD8^+^ T cells were isolated using CD4 and CD8 MicroBeads, respectively. The results showed that the protein levels of ATG7 were efficiently deleted in CD4^+^ (76%) and CD8^+^ (83%) cells in Atg7^f/f^CD4-Cre mice in comparison with Atg7^f/f^ mice (**Figure 4A**). Furthermore, ATG7 levels were detected in sham and CLP-induced Atg7^f/f^CD4-Cre and Atg7^f/f^ mice. As Atg7^f/f^CD4-Cre mice had a high mortality rate (40.91%) at 24 h after CLP, Atg7^f/f^ and Atg7^f/f^CD4-Cre mice were investigated at 18 h after CLP. Autophagy was observed by ATG7 protein levels, and Cyto-ID Green and Acridine orange staining for detecting autophagosomes/autolysosomes. For the ATG7 protein analyzed by Western blotting (**Figure 4B**), the amount of total protein loaded was double the amount of the blots in **Figure 4A** **,** in order to make the bands of Western blotting clearer for analyzing density. The results showed that ATG7 levels in CD4^+^ and CD8^+^ T cells were significantly decreased in sham Atg7^f/f^CD4-Cre mice compared to sham Atg7^f/f^ mice. ATG7 levels were also significantly decreased in CLP-induced Atg7^f/f^ and Atg7^f/f^CD4-Cre mice compared to their respective sham groups. However, a further decrease was found in CLP-induced Atg7^f/f^CD4-Cre mice when compared to CLP-induced Atg7^f/f^ mice (**Figure 4B**). Similar results were found by Cyto-ID Green and Acridine orange staining, showing that a further significant decrease in autophagy in Atg7^f/f^CD4-Cre mice after CLP compared to Atg7^f/f^ mice after CLP (**Figure 4C and 4D**).

### T cell-specific knockout of Atg7 increases mortality after CLP

**Figure 5:**
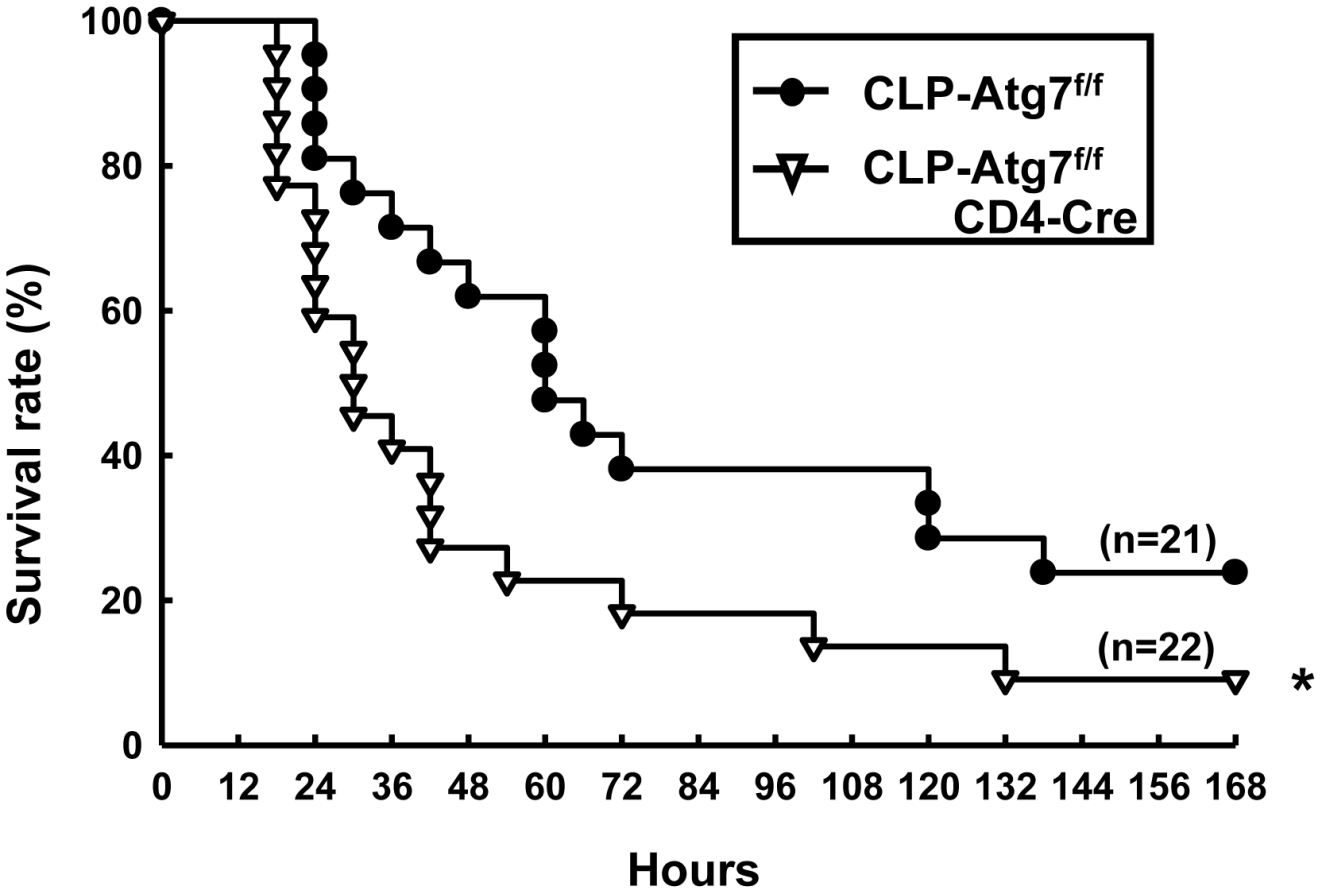
Impaired survival in CLP-induced Atg7^f/f^CD4-Cre mice. Atg7^f/f^ and Atg7^f/f^CD4-Cre mice underwent CLP surgery and survival was monitored for 7 days. Data were analyzed by the Kaplan-Meier method. CLP: cecal ligation and puncture. *P<0.05 vs. CLP-induced Atg7^f/f^ mice.

We further examined the survival of Atg7^f/f^ and Atg7^f/f^CD4-Cre mice after CLP. The results showed that Atg7^f/f^CD4-Cre mice exhibited significantly decreased survival at 7 days post CLP, when compared to CLP Atg7^f/f^ mice (**Figure 5**), suggesting that T cell specific inhibition of autophagy contributes to increased mortality in the CLP septic model.

### T cell-specific knockout of Atg7 results in a loss of CD4^+^CD8^−^ and CD4^−^CD8^+^ T cells after CLP

**Figure 6:**
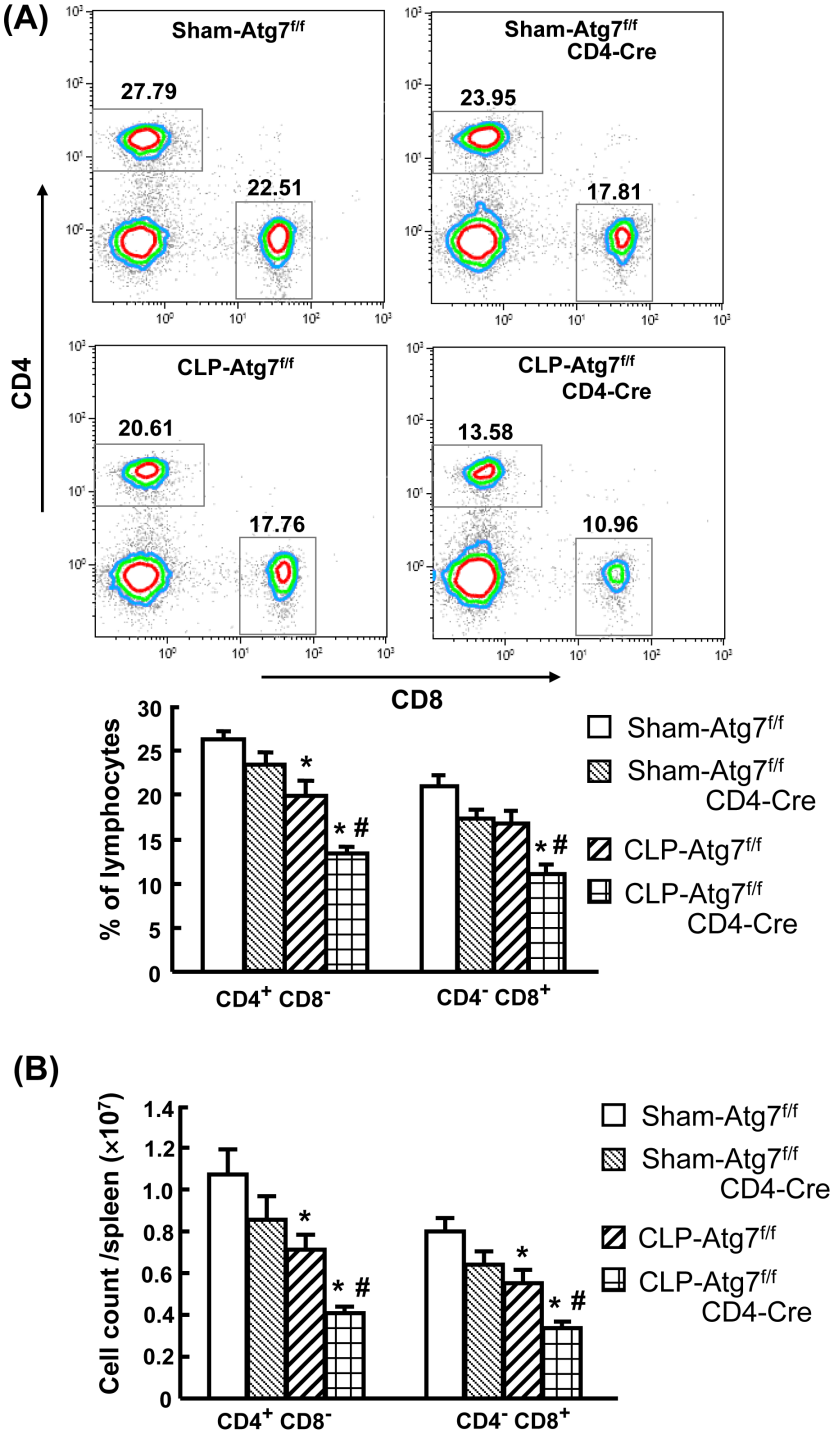
Impaired number of CD4^+^CD8^−^ and CD4^−^CD8^+^ T cells in CLP-induced Atg7^f/f^CD4-Cre mice. Splenocytes obtained at 18h after surgery were stained for surface markers (CD4 and CD8) and analyzed by flow cytometry. Viable lymphocytes were gated by using forward scatter versus side scatter (excluding cell debris). The gated lymphocytes were further gated on the CD4^+^CD8^−^ and CD4^−^CD8^+^ T cells. Representative histograms were shown percentage of lymphocytes (A). The absolute numbers of CD4^+^CD8^−^ and CD4^−^CD8^+^ T-cell subsets in the spleen were enumerated (B). Results obtained from 3 animals in each group are shown as mean ± SEM in the bar graph. Data are compared by one-way analysis of variance and Student-Newman Keul's test. CLP: cecal ligation and puncture. *P<0.05 vs. respective sham mice. #*P*<0.05 vs. CLP-induced Atg7^f/f^ mice.

Splenocytes obtained at 18 h after CLP were counted, stained for surface markers and analyzed by flow cytometry. The percentage and absolute number of CD4^+^CD8^−^ and CD4^−^CD8^+^ cells were evaluated. We found that both percentage and absolute number of CD4^+^CD8^−^ and CD4^−^CD8^+^ T cells were decreased in both Atg7^f/f^ and Atg7^f/f^CD4-Cre mice after CLP compared to respective shams (**Figure 6A and 6B**). However, CLP-induced Atg7^f/f^CD4-Cre mice showed a further significant decrease in CD4^+^CD8^−^ and CD4^−^CD8^+^ T cells compared to CLP-induced Atg7^f/f^ mice (**Figure 6A and 6B**).

### T cell-specific knockout of Atg7 leads to an increased susceptibility to apoptosis of CD4^+^CD8^−^ and CD4^−^CD8^+^ T cells after CLP

**Figure 7:**
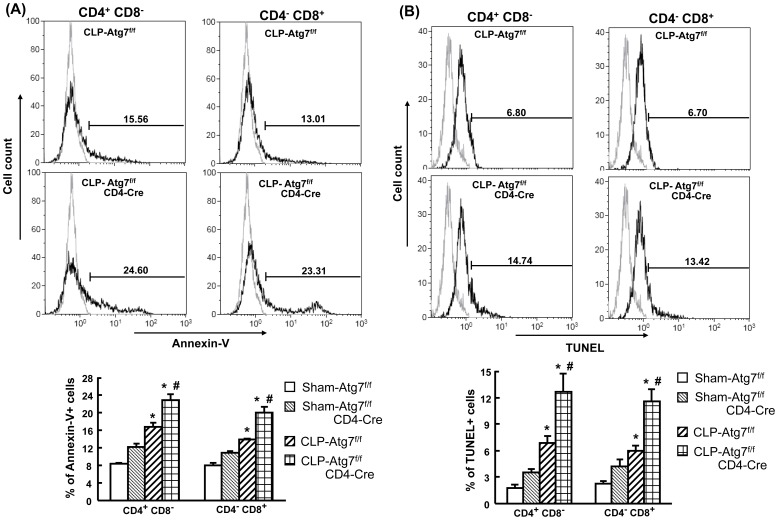
Increased apoptosis in CD4^+^CD8^−^ and CD4^−^CD8^+^ T cells in CLP-induced Atg7^f/f^CD4-Cre mice. Splenocytes obtained at 18h after surgery were stained for surface markers, Annexin-V (A) and TUNEL (B), and then analyzed by flow cytometry. Viable lymphocytes were gated by using forward scatter versus side scatter (excluding cell debris). The gated lymphocytes were further gated on the CD4^+^CD8^−^ and CD4^−^CD8^+^ T cells which were then analyzed for Annexin-V and TUNEL. Representative histograms of CLP-induced Atg7^f/f^ and Atg7^f/f^CD4-Cre mice were shown as percentage of CD4^+^CD8^−^ and CD4^−^CD8^+^ T cells that were positive for Annexin-V or TUNEL. The light gray histogram represents a staining control without adding Annexin-V or TUNEL. Results obtained from 4–6 animals in each group are shown as mean ± SEM in the bar graph. Data are compared by one-way analysis of variance and Student-Newman Keul's test. CLP: cecal ligation and puncture. *P<0.05 vs. respective sham mice. #*P*<0.05 vs. CLP-induced Atg7^f/f^ mice.

To further determine the percentage of apoptotic cells, splenocytes obtained at 18 h after CLP were stained for Annexin-V and TUNEL staining and analyzed by flow cytometry. As shown in **Figure 7A and 7B**, apoptosis induction was seen in CD4^+^CD8^−^ and CD4^−^CD8^+^ T cells in both Atg7^f/f^ and Atg7^f/f^CD4-Cre mice after CLP compared to respective sham mice. However, a further significant increase in apoptosis induction was observed in CLP Atg7^f/f^/CD4-Cre mice compared to CLP Atg7^f/f^ mice. This suggests that disrupted autophagy results in increased apoptosis in CD4^+^CD8^−^ and CD4^−^CD8^+^ T cells, accompanied by the loss of cell numbers after CLP.

Although it was noted in sham Atg7^f/f^CD4-Cre mice compared to sham Atg7^f/f^ mice that the cell count of CD4^+^CD8^−^ and CD4^−^CD8^+^ T cells was decreased and that T cell apoptosis was increased - these factors were not statistically significant.

### T cell-specific knockout of Atg7 decreases cytokine production of Th1, Th2 and Th17 after CLP

**Figure 8:**
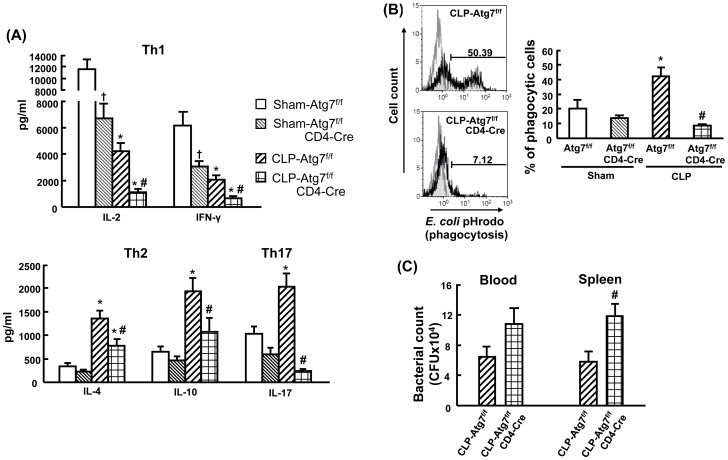
Decreased CD4^+^ cell cytokine production, macrophage phagocytosis and bacterial clearance in CLP-induced Atg7^f/f^CD4-Cre mice. Splenocytes obtained at 18h after surgery were isolated with CD4 MicroBeads, and stimulated by anti-CD3/CD28 for 24h for cytokine production (A). In vitro phagocytosis, splenocytes obtained at 18h after CLP and cultured with *E. coli* BioParticles for 1h (B). Cells were then stained with surface marker (F4/80) and analyzed by flow cytometry. The light gray histogram represents a staining control without adding *E. coli* BioParticles. Blood and spleen tissues were collected at 18h after CLP and analyzed for bacteria loads (C). For bacterial loads, results were expressed as CFU per milliliter of blood and CFU per spleen tissue. Values of cytokine production and bacterial loads are shown as mean ± SEM of 6–8 animals in each group. Values of phagocytosis are shown as mean ± SEM of 3 animals in each group. Data are compared by one-way analysis of variance and Student-Newman Keul's test for cytokine production and phagocytosis. Data are compared by two-tailed Student t-test for bacterial loads. CLP: cecal ligation and puncture. †*P*<0.05 vs. sham-operated Atg7^f/f^ mice. *P<0.05 vs. respective sham mice. #*P*<0.05 vs. CLP-induced Atg7^f/f^ mice.

To further determine the role of autophagy in regulation of T cell activity, we examined activation-induced cytokine production by CD4^+^ T cells. Splenic CD4^+^ T cells obtained at 18 h after CLP were isolated with CD4 MicroBeads, and then stimulated by anti-CD3/CD28 for 24 h. The cytokine production of Th1 (IL-2 and IFN-γ), Th2 (IL-4 and IL-10) and Th17 (IL-17) by CD4^+^ T cells were detected. As shown in **Figure 8A**, in sham groups autophagy-deficient CD4^+^ T cells isolated from Atg7^f/f^CD4-Cre mice had a significant decrease in IL-2 and IFN-γ production compared to Atg7^f/f^ mice. Moreover, decreased IL-2 and IFN-γ production, and increased IL-4 and IL-10 production were found in both Atg7^f/f^ and Atg7^f/f^CD4-Cre mice following CLP compared to their respective sham mice. However, CLP-induced autophagy-deficient CD4^+^ T cells showed a significant decrease in IL-2, IFN-γ, IL-4 and IL-10 production in comparison to CLP-induced Atg7^f/f^ mice. In additon, IL-17 production was significantly increased in CLP-induced Atg7^f/f^ mice compared to sham Atg7^f/f^ mice. However, IL-17 production had a dramatic decrease in CLP-induced autophagy-deficient CD4^+^ T cells when compared to the CLP-induced Atg7^f/f^ group. These findings indicate that disruption of autophagy in T cells results in inhibition of CD4^+^ T-cell activation after CLP.

### T cell-specific knockout of Atg7 decreases macrophage phagocytosis and bacterial clearance after CLP

As cytokine production by T cells mediates macrophage activation, we further analyzed phagocytotic activity in macrophages and bacterial loads in circulation and splenic tissues in Atg7^f/f^ and Atg7^f/f^CD4-Cre mice at 18 h after CLP. As shown in **Figure 8B**, we observed a significant increase in phagocytosis in Atg7^f/f^ but not in Atg7^f/f^CD4-Cre mice after CLP compared to their respective sham mice. Moreover, CLP-induced Atg7^f/f^CD4-Cre mice showed a significant decrease in phagocytosis compared to CLP-induced Atg7^f/f^ mice. These results were further confirmed by analyzing bacterial clearance. The results showed that CLP-induced Atg7^f/f^CD4-Cre mice had a significant increase in bacterial loads in the spleen compared to CLP-induced Atg7^f/f^ mice (**Figure 8C**). Systemic bacterial loads were also increased (but not significantly) in CLP-induced Atg7^f/f^CD4-Cre mice compared to CLP-induced Atg7^f/f^ mice (**Figure 8C**). This suggests that suppressed T-cell activation caused by disrupted autophagy in T cells results in decreased phagocytosis in macrophages and increased bacterial burden after CLP.

## Discussion

### Autophagy may play a protective role in the adaptive immune system via T cell apoptosis during sepsis

Although the role of autophagy in sepsis has been characterized in several organs - with previous studies from our group and others having reported protective effects against apoptotic cell death in liver, lung, heart and kidney in sepsis [10], [11], [12], [13], [14], [15] - its role in the regulation of the adaptive immune system is still to be ascertained. In this study we hypothesized that autophagy is involved in lymphocyte apoptosis during sepsis, as autophagy deficiency has been shown to induce apoptosis and decrease proliferation in T cells [16], [17].

Our study demonstrates that autophagy may play a protective role in the adaptive immune system during sepsis, with protection against cell apoptosis and an immunomodulatory role in T cells. This is the first study to investigate the role of autophagy in peripheral T lymphocytes in sepsis using a T cell specific autophagy knockout model, and demonstrated two significant findings. Firstly, we demonstrated that autophagy was down-regulated in CD4^+^ and CD8^+^ T cells in the late stage of sepsis. This was associated with increased apoptosis of CD4^+^ and CD8^+^ cells after sepsis. Secondly, elimination of the autophagy protein ATG7 with the use of T cell-specific *Atg7*-knockout mice resulted in an increase in sepsis-induced mortality and a loss of CD4^+^ and CD8^+^ T cells to apoptosis. This was accompanied by suppressed Th1/Th2/Th17 cytokine production by CD4^+^ T cells, reduced phagocytic activity in macrophages, and decreased bacterial clearance in the spleen in sepsis.

In the present study, autophagy was inhibited in CD4^+^ and CD8^+^ T cells at 18 h and 24 h after CLP. This finding is partially supported by Watanabe *et al* who found that the expression of the autophagic genes was slightly decreased in CD4^+^ T cells. However, they also noted that splenic TEM showed no difference in autophagosomes in CLP mice [35]. TEM alone may be insufficient, and therefore in our study a number of other parameters were assessed to support our findings, including the autophagy protein levels of LC3-II and ATG7, and autophagosome/autolysosome staining with Cyto-ID Green/acridine orange dye. Moreover, we demonstrated that mice lacking the essential autophagy gene *Atg7* in T lymphocytes were more susceptible to death after sepsis. These mice showed earlier onset of mortality than wild type mice and had overall increased mortality after sepsis, accompanied by loss of CD4^+^ and CD8^+^ T cells, and increased cellular apoptosis. These results indicated that sepsis led to down-regulation of autophagy in T lymphocytes, which may result in enhanced apoptosis induction and decreased survival in sepsis.

Although the ATG7 levels in *Atg7* deficient mice following CLP were dramatically decreased, the loss of CD4^+^ and CD8^+^ T cells was not absolute, with levels reduced by approximately 40% compared to CLP-induced Atg7^f/f^ mice. This suggests that the reduction in T cell numbers is only partially mediated by a deficiency in autophagy. Nonetheless this reduction in T cell number appears to be sufficient to exhibit clinically significant effects in T-cell-specific *Atg7*-knockout mice.

### Inhibition of autophagy may block T cell activation in sepsis

Autophagy, as well as being regulated by cytokines, can itself directly influence the transcription and secretion of a number of cytokines. It has been reported that plasmacytoid dendritic cells deficient in *Atg5*, or treated with autophagy inhibitors, failed to produce IFN-α in response to viral infection [24]. Moreover, T cells deficient in *Atg7*, or treated with lysosome inhibitors for blocking autophagy showed profound defects in their proliferative responses and their ability to produce cytokines, IL-2 and IFN-γ, after anti-CD3 and anti-CD28 stimulation, indicating that blockade of autophagy inhibited T cell activation [36]. This effect was further demonstrated, at least in part, due to the defects in energy metabolism caused by autophagy blockade [36]. In the present study we demonstrated that mice with disruption of *Atg7* in T cells exhibited a reduced cytokine production of IL-2, IFN-γ, IL-4, IL-10 and IL-17 by CD4^+^ T cells after sepsis, compared to septic wild-type mice. This suggests that the deregulation of autophagy contributes to sepsis suppressed T cell immune function, but the exact mechanism of suppressed cytokine production remains to be ascertained. Suppressed cytokine production may be mediated by defective mRNA transcription rather than defective secretion, as mRNA expression of two cytokines (IFN-γ and IL-10) were decreased in autophagy-deficient CD4^+^ cells after sepsis (unpublished data). Moreover, autophagy has been shown to play a critical role in maintaining energy homeostasis, and it is known that T cells require increased production of energy to sustain cell growth, activation and cytokine release [36], [37]. Suppressed cytokine production of Th1/Th2/Th17 by *Atg7*-deficient CD4^+^ T cells after sepsis may therefore be partially mediated by insufficient energy secondary to autophagy deficiency. Nonetheless, this needs to be further clarified.

### Autophagy may be involved in both the adaptive and innate immune response in sepsis

Although innate immunity plays a central role in response to bacterial infection and clearance, there is increasing evidence showing that components of adaptive immunity, in particular various T cell populations, provide critical signals to perpetuate ongoing innate anti-bacterial responses. Th1 and Th17 effector cells have been shown to activate macrophages to kill ingested pathogens [38], [39]. IFN-γ secreted by CD4 T cells has also been indicated to activate macrophages to phagocyte and digest intracellular bacteria [40], [41]. Therefore, one possibility is that suppression of T cell immune responses, including suppressed IFN-γ production, can result in an increase in bacterial burden after infection. This hypothesis is supported by our finding that mice with disrupted autophagy in T cells had decreased phagocytic capacity in macrophages and decreased bacterial clearance in the spleen after sepsis.

In conclusion, T cell specific inhibition of autophagy results in decreased survival, increased T cell immunosuppression, apoptosis, cell death and reduced macrophage phagocytosis, as well as elevated splenic bacterial loads after sepsis. This suggests that autophagy plays a protective role against sepsis-induced T lymphocyte apoptosis and immunosuppression. Novel future treatment strategies in severe clinical sepsis may therefore include autophagy enhancers.
